# Beyond the Headache: Sleep-Related Symptom Burden in Children and Adolescents with Migraine and Episodic Tension-Type Headache

**DOI:** 10.3390/children13101357

**Published:** 2026-10-06

**Authors:** Veli Yildirim, Aynur Özge, Recep Dokuyucu

**Affiliations:** 1 Department of Child Psychiatry, Special Clinic, 33110 Mersin, Türkiye; drveliyildirim@gmail.com; 2 Department of Neurology, Faculty of Medicine, Mersin University, 33110 Mersin, Türkiye; aynurozge@gmail.com; 3 Department of Physiology, Medical Specialization Training Center (TUSMER), 06230 Ankara, Türkiye

**Keywords:** pediatric headache, migraine, episodic tension-type headache, sleep-related symptoms, Pediatric Sleep Questionnaire, behavioral problems, sleep screening

## Abstract

**Highlights:**

**What are the main findings?**

- Clinically relevant sleep-related symptoms were more frequent in participants with primary headache disorders than in healthy controls (58.3% vs. 27.7%).
- Both migraine and episodic tension-type headache were associated with higher PSQ total and Behavioral Problems scores, with no significant difference between the two headache diagnoses.
- The excess symptom burden involved awakening/developmental and behavioral domains rather than snoring symptoms.

**What are the implications of the main findings?**

- Sleep screening should be incorporated into the routine assessment of children and adolescents with both migraine and episodic tension-type headache.
- Assessment should extend beyond snoring and sleep-disordered breathing to include awakening difficulties, daytime symptoms, and behavioral problems.
- Positive questionnaire findings should prompt further clinical evaluation because the PSQ is a screening instrument and does not establish a specific sleep-disorder diagnosis.

**Abstract:**

Background/Objectives: Sleep disturbances are frequently reported in children with primary headache disorders, but differences in sleep-related symptom profiles between pediatric migraine and episodic tension-type headache (TTH) remain unclear. This study aimed to compare Pediatric Sleep Questionnaire (PSQ) total and domain scores among children and adolescents with migraine, episodic TTH, and healthy controls and to examine whether these patterns differed according to sex and age. Methods: This analytical cross-sectional study included 197 participants aged 6–18 years recruited in Mersin, Türkiye, between 1 March 2022 and 31 December 2023. Primary headache disorders were diagnosed according to the International Classification of Headache Disorders, third edition. The headache group comprised 76 patients with migraine and 27 with episodic TTH, while 94 healthy students constituted the control group. Parents completed the validated Turkish extended PSQ. A PSQ total score ≥ 6 was considered indicative of clinically relevant sleep-related symptoms. Headache characteristics and intensity, measured using a 0–10 numerical rating scale, were also recorded. Results: A PSQ total score ≥ 6 was observed in 60/103 participants with headache (58.3%) and 26/94 healthy controls (27.7%; *p* < 0.001). Compared with controls, participants with headache had higher median PSQ total scores (6 [interquartile range, 4–8] vs. 4 [2–6]; *p* < 0.001), Breathing Problems scores (2 [1–3] vs. 1.5 [1–3]; *p* = 0.022), Awakening and Developmental Symptoms scores (2 [1–2] vs. 1 [0–2]; *p* < 0.001), and Behavioral Problems scores (1 [0–3] vs. 0 [0–1]; *p* < 0.001). Snoring Problems scores did not differ (p = 0.659). Both migraine and episodic TTH groups had higher PSQ total and Behavioral Problems scores than healthy controls, without significant differences between the two headache diagnoses. Compared with episodic TTH, migraine was associated with a longer headache history (*p* = 0.011), more frequent attacks (*p* = 0.021), and greater headache intensity (*p* = 0.013). Conclusions: Children and adolescents with migraine or episodic TTH exhibit a substantially greater burden of clinically relevant sleep-related symptoms than healthy controls. This burden is primarily reflected in overall, awakening/developmental, and behavioral symptoms rather than snoring. Sleep screening should therefore be incorporated into the clinical assessment of both pediatric migraine and episodic TTH.

## 1. Introduction

Primary headache disorders are among the most common neurological conditions in children and adolescents. A recent systematic review and meta-analysis estimated pooled prevalence rates of approximately 11% for migraine and 17% for tension-type headache (TTH) in pediatric populations, although substantial heterogeneity was observed across studies [1]. Migraine and TTH may impair school attendance, academic performance, social participation, emotional well-being, and overall quality of life [2]. Their differentiation can be particularly challenging in childhood because headache characteristics may be less typical than those observed in adults and some clinical features may overlap [3]. Moreover, the epidemiology of pediatric headache changes across development: migraine prevalence is relatively similar between boys and girls in childhood but increases markedly among girls during adolescence [4].

Sleep disturbances and primary headaches have a complex and potentially bidirectional relationship. Insufficient or fragmented sleep may precipitate or exacerbate headache attacks, whereas recurrent headaches may interfere with sleep initiation, continuity, and restorative quality [5,6]. This interaction may partly reflect shared involvement of hypothalamic and brainstem networks and common neurochemical systems, including melatonin, orexin, adenosine, and calcitonin gene-related peptide [5]. Children with primary headaches have been reported to experience difficulties initiating or maintaining sleep, night awakenings, daytime sleepiness, parasomnias, bruxism, snoring, and symptoms suggestive of sleep-disordered breathing more frequently than healthy children [7,8,9]. Although these associations have been studied most extensively in pediatric migraine, sleep-related symptoms have also been reported in children with TTH. However, findings regarding differences between migraine and TTH remain inconsistent, partly because of small samples, heterogeneous headache classifications, referral-based populations, and differences in the instruments used to assess sleep [10,11,12].

Sleep-related symptoms may also be accompanied by behavioral and cognitive difficulties. Sleep fragmentation and daytime sleepiness can manifest as irritability, impaired attention, hyperactivity, impulsivity, and reduced academic functioning, which may complicate the clinical assessment of children with recurrent headaches. Previous research has demonstrated that children with persistent headaches may have poorer sleep quality, greater daytime sleepiness, and more behavioral problems than healthy controls, with possible differences according to sex [13]. Therefore, an assessment limited to nocturnal symptoms may underestimate the broader functional consequences of disturbed sleep in this population.

The Pediatric Sleep Questionnaire (PSQ) includes parent-reported scales addressing sleep-related breathing symptoms, snoring, sleepiness, and behavioral concerns [14]. The extended Turkish version used in the present study was evaluated by Öner et al. [15]. Although the PSQ is a screening instrument rather than a diagnostic substitute for clinical evaluation or polysomnography, its multidimensional structure may facilitate the identification of clinically relevant sleep and behavioral symptom patterns in children with headache disorders. Nevertheless, comparative evidence evaluating PSQ-defined symptom profiles across migraine, episodic TTH, and healthy children remains limited, particularly with respect to age- and sex-related variation.

Accordingly, this study aimed to compare the frequency and pattern of parent-reported sleep-related and behavioral symptoms, as well as PSQ total and domain scores, among children and adolescents with migraine, those with episodic TTH, and healthy school controls. Secondary objectives were to investigate whether these symptom patterns differed according to sex and age.

## 2. Materials and Methods

### 2.1. Study Design and Setting

This analytical cross-sectional study included children and adolescents aged 6–18 years with migraine or episodic tension-type headache (TTH), together with a healthy control group. Patients with primary headache disorders were recruited from the Pediatric Neurology Outpatient Clinic of Mersin University School of Medicine Hospital, Mersin, Türkiye, between 1 March 2022 and 31 December 2023. Healthy controls were recruited from students in the sixth, seventh, and eighth grades at Mezitli Public School in Mersin. Neurological examinations, headache assessments, and questionnaire administration were completed during a single study visit.

A total of 197 participants were included: 103 patients with a primary headache disorder and 94 healthy controls. The headache group comprised 76 patients with migraine and 27 patients with episodic TTH. The migraine group included patients with migraine with aura, migraine without aura, and chronic migraine. The TTH group included only patients with an episodic form of the disorder.

### 2.2. Eligibility Criteria

Children and adolescents were eligible for the headache group if they were 6–18 years of age and fulfilled the International Classification of Headache Disorders, 3rd edition (ICHD-3) diagnostic criteria for migraine or episodic TTH [16]. Additional eligibility criteria were the absence of current or previous psychotropic medication use and the absence of regular daily analgesic or migraine-specific medication use at the time of assessment.

Patients were excluded if they had a secondary headache disorder, simultaneously fulfilled the diagnostic criteria for both migraine and TTH, or had a history of a severe concussion, another major neurological disorder, a rheumatologic disease, or a malignant disorder.

Healthy controls were required to be 6–18 years of age and to have no history of recurrent headache, diagnosed neurological or psychiatric disease, or regular medication use. Participants in the control group were evaluated using the same demographic form and sleep questionnaire as the headache groups.

### 2.3. Headache Assessment and Classification

All patients underwent a comprehensive neurological evaluation conducted by a headache specialist. Migraine and episodic TTH diagnoses were established according to the ICHD-3 diagnostic criteria [16]. Patients were classified into mutually exclusive diagnostic groups. Migraine was further categorized as migraine with aura, migraine without aura, or chronic migraine.

A standardized headache intake questionnaire was used to record headache location, pain quality, headache intensity, attack duration, associated symptoms, and attack frequency. The duration of the headache disorder was recorded in months, attack duration in hours, and attack frequency as the number of attacks during the preceding month. Usual headache intensity was self-reported by the participant using an 11-point numerical rating scale (NRS-11), ranging from 0 (“no pain”) to 10 (“the worst pain imaginable”). Participants were asked to rate the typical intensity of their headache attacks rather than the intensity of pain experienced at the time of assessment. Age-appropriate standardized instructions were provided to younger children when necessary, and parental assistance was limited to clarifying the clinical context and was not used to replace the child’s subjective pain rating.

### 2.4. Pediatric Sleep Questionnaire

Sleep-related and behavioral symptoms were assessed using the parent-completed extended version of the Pediatric Sleep Questionnaire (PSQ). The original PSQ was developed to evaluate sleep-related breathing symptoms, snoring, daytime sleepiness, and associated behavioral difficulties in children [14]. The extended Turkish version was validated by Öner et al. and demonstrated acceptable internal consistency, with a Cronbach’s alpha coefficient of 0.77 for the overall questionnaire [15]. The extended Turkish form was selected to assess symptoms across several sleep and behavioral domains. Its validation sample did not consist of children with primary headache disorders; consequently, PSQ scores and the ≥6 threshold were interpreted as screening findings rather than diagnostic measures in this cohort.

Although parents completed the extended questionnaire, the PSQ total and factor scores were calculated from the selected items specified in the Turkish validation study [15]. Responses to other questionnaire items were not assumed to represent separately validated sleep-disorder outcomes. The extended Turkish PSQ contains 70 items and includes four factor-derived symptom domains: Snoring Problems, Behavioral Problems, Breathing Problems, and Other Problems [15]. The Behavioral Problems domain includes items addressing inattention, hyperactivity, and impulsivity. The Other Problems domain includes symptoms such as difficulty awakening in the morning, morning headache, unrefreshing sleep, and developmental concerns. For greater clinical clarity, this domain was referred to as the “Awakening and Developmental Symptoms” (ADS) domain in the present study. This terminological modification did not alter the original item content, responses, or scoring.

Items were answered as “yes,” “no,” or “don’t know.” Positive responses were scored as 1, negative responses as 0, and “don’t know” responses were treated as missing rather than assigned a numerical symptom score. Domain scores and the PSQ total score were calculated by summing the positively endorsed items included in the corresponding scoring set. Higher scores indicated a greater burden of parent-reported sleep-related or behavioral symptoms. In accordance with the validation study of the extended Turkish PSQ, a total score of ≥6 was used to identify participants with clinically relevant sleep-related symptoms [15]. The PSQ was used as a screening instrument and was not considered a substitute for clinical sleep assessment or polysomnography. The questionnaire was completed by a parent or legal guardian. Adolescents were permitted to assist their parents in responding when clarification regarding subjective symptoms was required.

### 2.5. Study Outcomes

The primary outcomes were differences in PSQ total and domain scores (Snoring Problems, Breathing Problems, Behavioral Problems, and Awakening and Developmental Symptoms [ADS]) among participants with migraine, episodic TTH, and healthy controls.

Secondary outcomes were the proportion of participants with a PSQ total score ≥ 6, sex-stratified differences in PSQ scores, and differences in headache duration, attack duration, monthly attack frequency, and headache intensity between the migraine and episodic TTH groups. Exploratory analyses examined associations between age and PSQ scores according to headache diagnosis and sex. Additional exploratory outcomes were usual daytime napping according to diagnostic group and the proportion of PSQ-positive screens according to school-calendar period within the headache cohort.

### 2.6. Statistical Analysis

Statistical analyses were performed using SPSS version 27.0 (SPSS Inc., Chicago, IL, USA). Continuous variables were examined for distributional characteristics before analysis. Normally distributed variables were summarized as mean ± standard deviation, whereas non-normally distributed variables were reported as median and interquartile range (25th–75th percentiles). Categorical variables were presented as numbers and percentages. For two-group comparisons, normally distributed continuous variables were analyzed using the independent-samples *t*-test, and non-normally distributed variables were analyzed using the Mann–Whitney *U* test. Comparisons of normally distributed variables among migraine, episodic TTH, and healthy control groups were performed using one-way analysis of variance. Non-normally distributed variables and PSQ domain scores were compared among the three groups using the Kruskal–Wallis test. When an omnibus test was statistically significant, pairwise post hoc comparisons were performed with Bonferroni adjustment. Categorical variables were compared using Pearson’s chi-square test or Fisher’s exact test, as appropriate. Sex-stratified comparisons of PSQ scores among the migraine, episodic TTH, and healthy control groups were performed using the Kruskal–Wallis test. Associations between age and PSQ scores were examined using Spearman’s rank-correlation coefficient and were explored separately according to headache type and sex. Two separate sensitivity analyses were performed. First, PSQ total scores and the proportion of participants with a PSQ total score ≥ 6 were compared after restricting the headache groups to the age range observed among healthy controls. Second, the same outcomes were compared after excluding patients classified as having chronic migraine from the original study sample. Usual daytime napping, assessed using PSQ item B5, was compared among the three diagnostic groups using Pearson’s chi-square test, followed by Bonferroni-adjusted pairwise Fisher’s exact tests. Among patients with headache, study visits were classified as occurring during a school term or a scheduled break according to the school calendar applicable to the visit date. PSQ-positive proportions were compared between these periods using Fisher’s exact test; an odds ratio and 95% confidence interval were calculated. All tests were two-sided, and a *p* value < 0.05 was considered statistically significant.

## 3. Results

**Figure 1:**
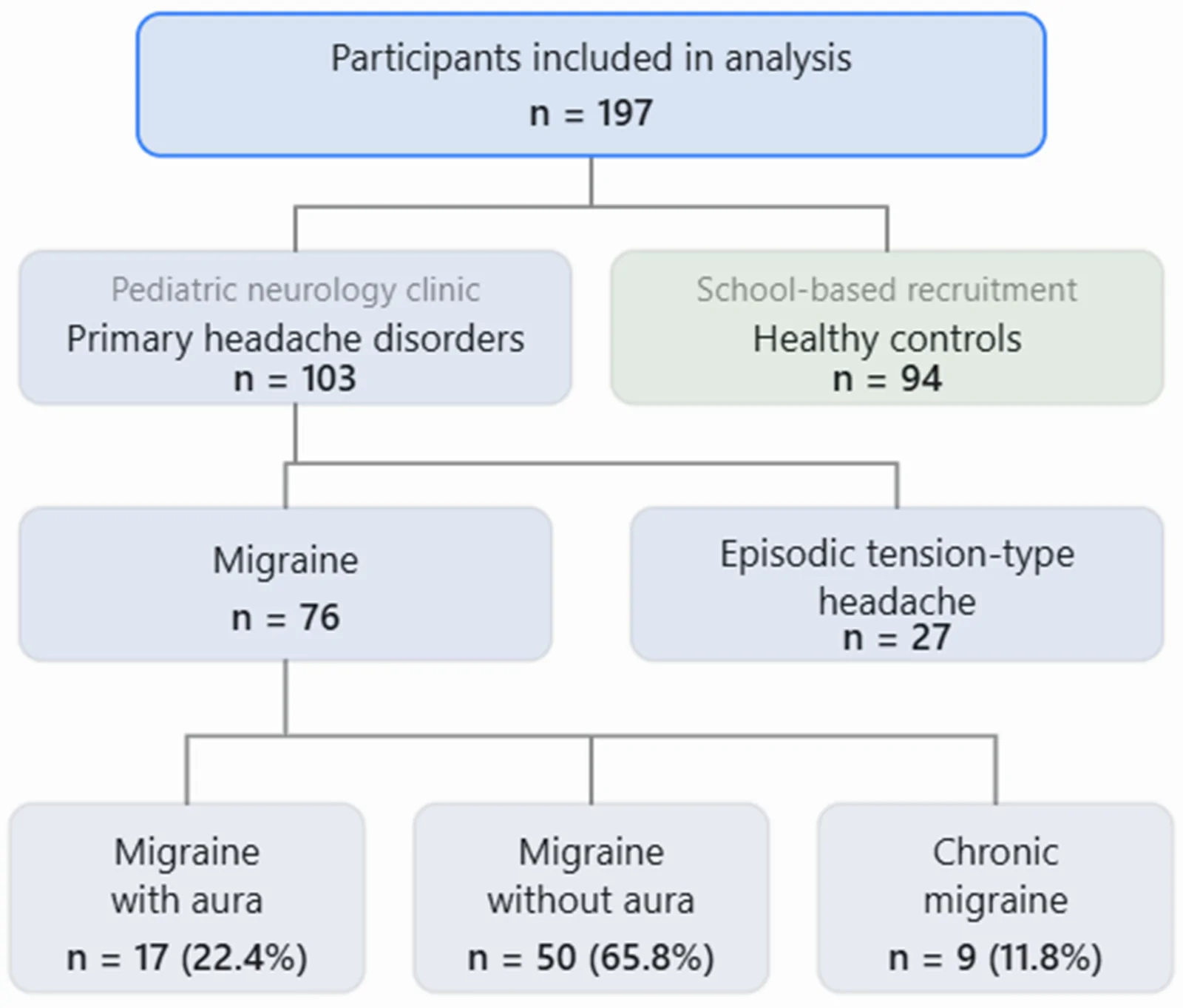
Flow diagram of participant recruitment and diagnostic classification.

This study included 197 participants: 103 patients with a primary headache disorder and 94 healthy controls (Figure 1). Among the patients with headache, 76 were diagnosed with migraine and 27 with episodic tension-type headache (TTH). The migraine group comprised 17 patients (22.4%) with migraine with aura, 50 (65.8%) with migraine without aura, and 9 (11.8%) with chronic migraine. All patients in the TTH group had episodic TTH (Figure 1).

The demographic characteristics and Pediatric Sleep Questionnaire (PSQ) results are presented in Table 1. The mean age was comparable between the headache and healthy control groups (13.1 ± 2.44 vs. 13.1 ± 1.00 years, respectively; *p* = 0.874). Female participants were more frequent in the headache group than in the healthy control group (66.0% vs. 47.9%; *p* = 0.010). A PSQ total score ≥ 6, indicating clinically relevant sleep-related symptoms, was observed in 60 of 103 participants with primary headache disorders (58.3%) and 26 of 94 healthy controls (27.7%; *p* < 0.001). Compared with healthy controls, participants with primary headache disorders had higher median PSQ total scores (6 [IQR, 4–8] vs. 4 [IQR, 2–6]; *p* < 0.001), Breathing Problems scores (2 [IQR, 1–3] vs. 1.5 [IQR, 1–3]; *p* = 0.022), Awakening and Developmental Symptoms scores (2 [IQR, 1–2] vs. 1 [IQR, 0–2]; *p* < 0.001), and Behavioral Problems scores (1 [IQR, 0–3] vs. 0 [IQR, 0–1]; *p* < 0.001). Snoring scores did not differ significantly between the groups (*p* = 0.659) (Table 1).

In the age-range-restricted sensitivity analysis, 16 patients with migraine and 5 with episodic TTH were excluded, leaving 60 patients with migraine, 22 with episodic TTH, and 94 controls. A PSQ total score ≥ 6 was recorded in 47/82 patients with headache (57.3%) and 26/94 controls (27.7%; odds ratio, 3.51; 95% CI, 1.87–6.59). Detailed results of the age-range-restricted sensitivity analysis are presented in Supplementary Table S1.

**Figure 2:**
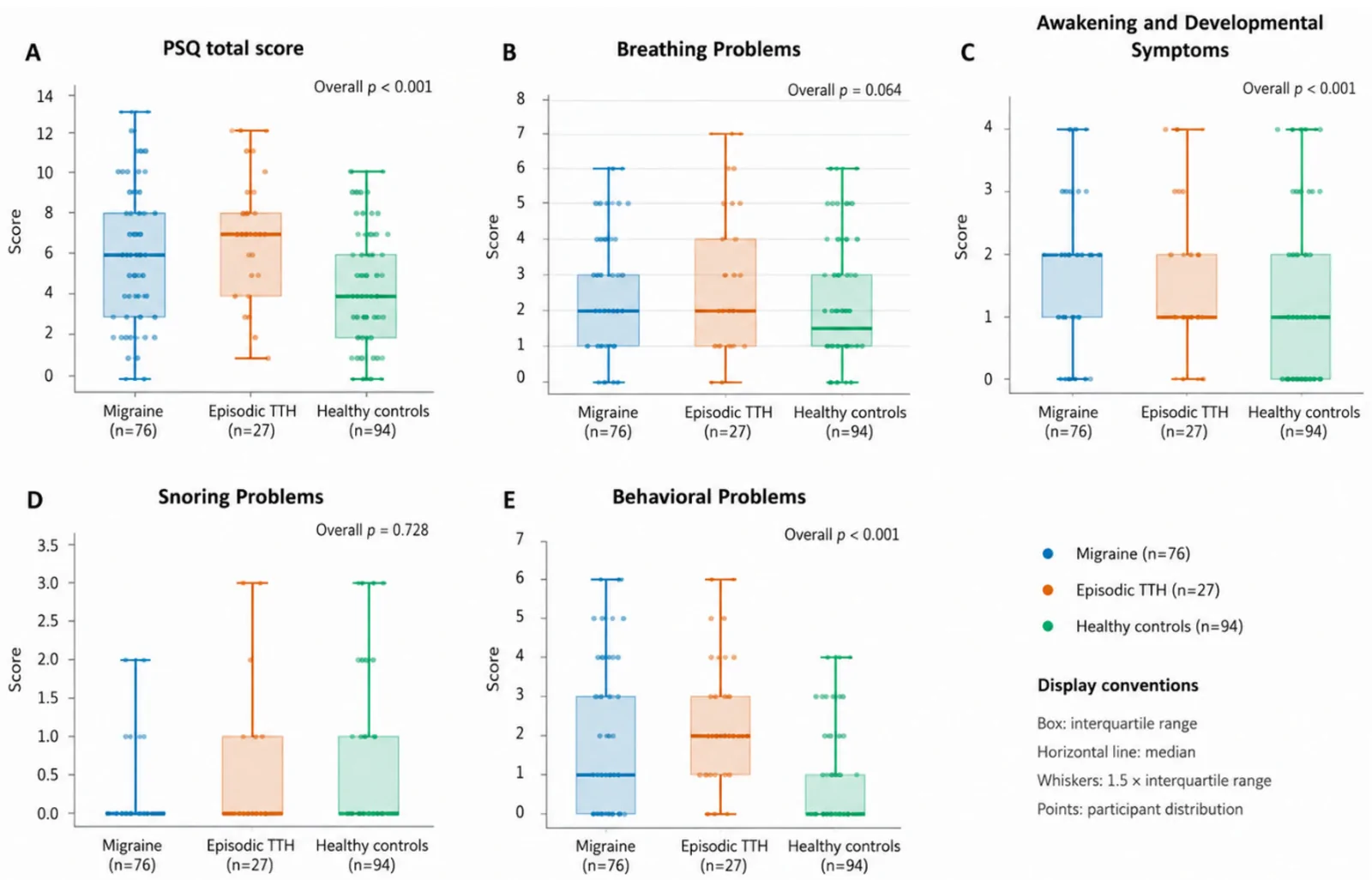
Distribution of Pediatric Sleep Questionnaire total and domain scores among the migraine, episodic tension-type headache, and healthy control groups. Box-and-jitter plots show the distributions of the PSQ total score and the Breathing Problems, Awakening and Developmental Symptoms, Snoring Problems, and Behavioral Problems domain scores. Boxes represent the interquartile range, horizontal lines indicate medians, and individual points represent participants.

Age did not differ significantly among the migraine, episodic TTH, and healthy control groups (*p* = 0.499). The proportions of female participants were 64.5%, 70.4%, and 47.9%, respectively (*p* = 0.032). PSQ total scores differed significantly among the three groups (*p* < 0.001). Median PSQ total scores were 6 (IQR, 3–8) in the migraine group, 7 (IQR, 4–8) in the episodic TTH group, and 4 (IQR, 2–6) in the healthy control group. Post hoc comparisons showed higher PSQ total scores in both headache groups than in healthy controls, whereas the migraine and episodic TTH groups did not differ significantly from each other. Behavioral Problems scores also differed among the groups (*p* < 0.001), with higher scores in both the migraine and episodic TTH groups than in healthy controls. ADS scores differed significantly overall (*p* < 0.001); post hoc analysis demonstrated a higher score in the migraine group than in healthy controls. No significant differences were detected among the groups in Breathing Problems (*p* = 0.064) or Snoring Problems scores (*p* = 0.728) (Table 2 and Figure 2).

After exclusion of the nine patients with chronic migraine, the sensitivity analysis included 67 patients with non-chronic migraine, 27 with episodic TTH, and 94 healthy controls. Median PSQ total scores were 6 (IQR, 3–8), 7 (IQR, 4–8), and 4 (IQR, 2–6), respectively (overall *p* < 0.001). In Bonferroni-adjusted pairwise comparisons, both headache groups had higher PSQ total scores than controls (both adjusted *p* < 0.001), whereas the two headache groups did not differ significantly (adjusted *p* = 1.000). A PSQ total score ≥ 6 was observed in 38/67 patients with non-chronic migraine (56.7%), 16/27 with episodic TTH (59.3%), and 26/94 controls (27.7%; overall *p* < 0.001). Detailed results are presented in Supplementary Table S2.

Patients with migraine had a longer history of headache than those with episodic TTH (27.0 ± 27.6 vs. 12.7 ± 12.1 months; *p* = 0.011). The mean duration of individual attacks did not differ significantly between the migraine and episodic TTH groups (7.3 ± 8.6 vs. 5.9 ± 10.4 h; *p* = 0.557). Migraine patients reported more frequent attacks during the preceding month than patients with episodic TTH (11.1 ± 9.6 vs. 7.2 ± 6.3 attacks/month; *p* = 0.021). Usual headache intensity measured using the 0–10 numerical rating scale was also higher in the migraine group (6.9 ± 1.6 vs. 6.0 ± 1.8; *p* = 0.013) (Table 3).

Among female participants, ADS (*p* = 0.030), Behavioral Problems (*p* < 0.001), and PSQ total scores (*p* = 0.003) differed significantly among the migraine, episodic TTH, and healthy control groups. Post hoc analyses showed higher Behavioral Problems and PSQ total scores in both headache groups than in healthy controls. ADS scores were higher in female patients with migraine than in female healthy controls. No significant group differences were observed in Breathing Problems (*p* = 0.149) or Snoring Problems scores (*p* = 0.712). Among male participants, ADS (*p* = 0.024), Behavioral Problems (*p* = 0.040), and PSQ total scores (*p* = 0.020) differed significantly among the three groups. Post hoc comparisons showed a higher ADS score in male patients with migraine than in male healthy controls, a higher Behavioral Problems score in male patients with episodic TTH than in male healthy controls, and higher PSQ total scores in both headache groups than in healthy controls. Breathing Problems (*p* = 0.734) and Snoring Problems scores (*p* = 0.908) did not differ significantly among the male groups (Table 4).

**Figure 3:**
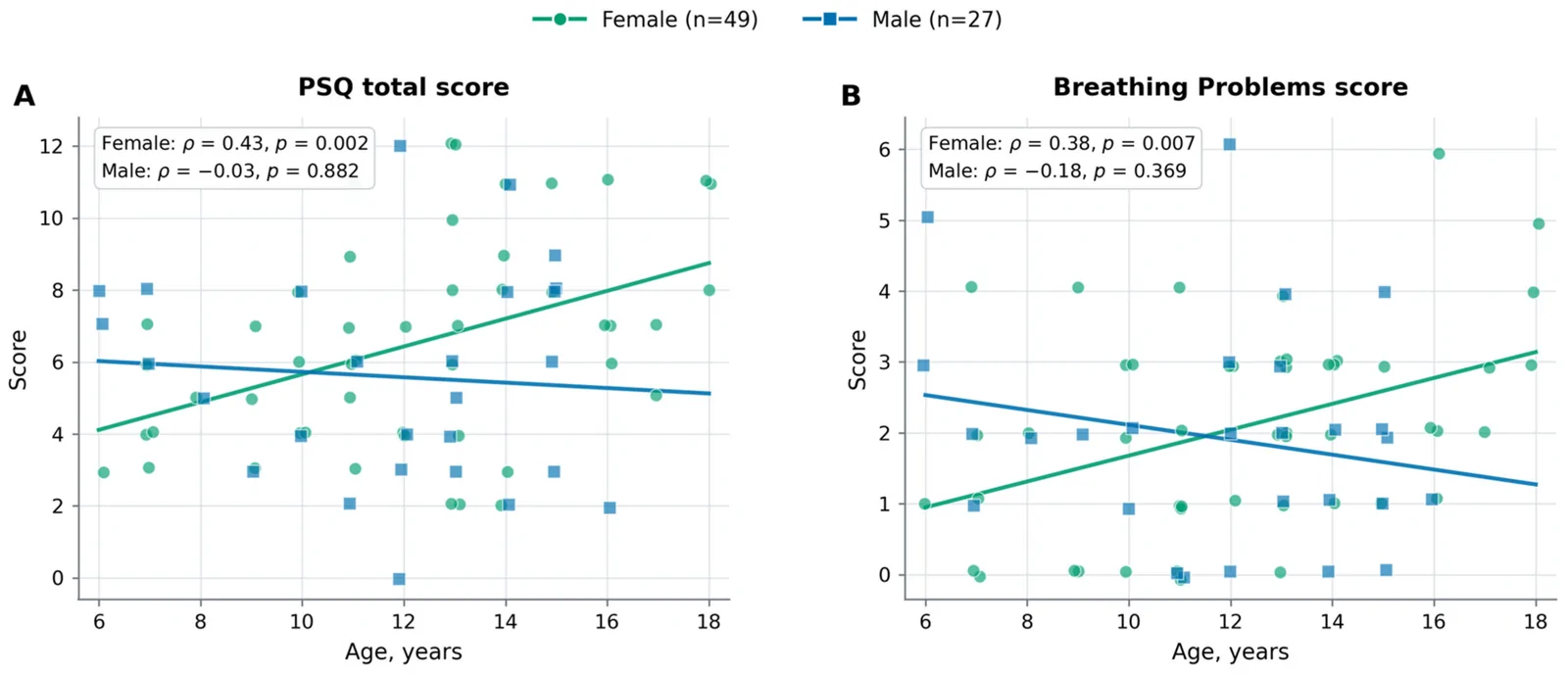
Sex-stratified associations of age with Pediatric Sleep Questionnaire scores in patients with migraine. Panel (**A**) shows the association between age and the PSQ total score. Panel (**B**) shows the association between age and the Breathing Problems score. Individual observations are presented separately for female and male participants; Spearman’s rank-correlation coefficients and corresponding *p* values are displayed within each panel.

In exploratory sex-stratified analyses, age was positively correlated with both the PSQ total score (Spearman’s ρ = 0.43, *p* = 0.002) and the Breathing Problems score (Spearman’s ρ = 0.38, *p* = 0.007) among female patients with migraine. In contrast, no significant correlations were observed among male patients with migraine for either the PSQ total score (Spearman’s ρ = −0.03, *p* = 0.882) or the Breathing Problems score (Spearman’s ρ = −0.18, *p* = 0.369). Age was also not significantly correlated with these PSQ scores in female or male patients with episodic TTH (all *p* > 0.05) (Figure 3).

In an exploratory item-level analysis, usual daytime napping was reported for 26/76 participants with migraine (34.2%), 7/27 with episodic TTH (25.9%), and 11/94 healthy controls (11.7%; overall *p* = 0.002). After Bonferroni adjustment, napping was reported more frequently in the migraine group than in controls (adjusted *p* = 0.002); the other pairwise comparisons were not statistically significant (Supplementary Table S3).

In an exploratory comparison by school-calendar period, 70 patients with headache were assessed during a school term and 33 during a scheduled break. A PSQ total score ≥ 6 was observed in 44/70 (62.9%) and 16/33 (48.5%), respectively (odds ratio, 1.80; 95% CI, 0.78–4.15; Fisher’s exact *p* = 0.201). The corresponding proportions were 33/52 (63.5%) versus 11/24 (45.8%) in migraine and 11/18 (61.1%) versus 5/9 (55.6%) in episodic TTH (Supplementary Table S4).

## 4. Discussion

In the present study, a PSQ total score ≥ 6 was observed in 58.3% of children with primary headache disorders and 27.7% of healthy controls, an absolute difference of 30.6 percentage points. The headache group also had a higher median PSQ total score (6 vs. 4), although the score distributions overlapped. These results indicate a greater burden of parent-reported sleep-related symptoms in the clinic-based headache group; a positive PSQ screen does not establish a sleep-disorder diagnosis.

This overall pattern is consistent with Bruni et al., who reported more frequent sleep disturbances in children with headache than in healthy controls [17]. Açıkel et al. likewise identified sleep disturbances in children with migraine and TTH [18], while Voci et al. found that sleep disturbances were common among children with migraine [8]. Because these studies used different sleep measures and headache populations, their reported frequencies should not be directly compared with the PSQ screening proportion in our study.

Both the migraine and episodic TTH groups had higher PSQ total scores than controls, with no significant difference between the two headache groups. Bruni et al. similarly found no significant overall difference in sleep disturbance between children with migraine and TTH [17]. In contrast, Cheraghi et al. reported poorer sleep quality in the migraine group [19], whereas Rabner et al. observed greater sleep disturbance in children with TTH and new daily persistent headache than in those with migraine [13]. Differences in headache chronicity, clinical setting, and sleep assessment may contribute to these varying results. The small episodic TTH group and the absence of a chronic TTH group limit conclusions about diagnostic differences; a nonsignificant comparison does not establish equivalence. After exclusion of the nine patients with chronic migraine, both the non-chronic migraine and episodic TTH groups continued to show higher PSQ total scores than controls, while no significant difference was detected between the two headache groups. This suggests that the observed pattern was not driven solely by the inclusion of chronic migraine. The small episodic TTH group and absence of a chronic TTH group limit conclusions about headache chronicity or equivalence between diagnoses.

In our cohort, patients with migraine had a longer headache history, more frequent attacks, and greater headache intensity than those with episodic TTH. We did not, however, analyze whether these headache characteristics were associated with PSQ scores. In other pediatric migraine cohorts, Miller et al. linked attack frequency and duration to specific sleep disturbances [20]. Heyer et al. found associations between headache characteristics and sleep disturbance using prospective diary data [21]. Voci et al. associated sleep disturbance with migraine frequency, attack severity, and acute treatment efficacy [8]. These studies provide context, but our data cannot establish whether headache characteristics and sleep symptoms were related within our cohort.

Usual daytime napping was reported by 34.2% of participants with migraine, 25.9% with episodic TTH, and 11.7% of controls; only the migraine–control difference remained statistically significant after adjustment (adjusted *p* = 0.002). Aaltonen et al. reported sleep coincided with the resolution of some migraine attacks in children [22]. However, PSQ item B5 records usual napping and does not establish whether a nap occurred during an attack, followed acute treatment, or relieved pain. The reason for the reported naps therefore cannot be determined from this item.

The combined headache group had a higher median ADS score than controls (2 vs. 1), with the clearest diagnostic-group difference observed between migraine and controls. Gilman et al. also documented insufficient sleep, difficulty initiating sleep, night waking, and daytime sleepiness in adolescents with primary headache [23]. However, the one-point median difference and overlapping score distributions leave its clinical importance for an individual child uncertain. ADS is a descriptive label for the Turkish PSQ’s “Other Problems” factor, which showed limited reliability in the validation study [15]. Because this factor includes a morning-headache item, the higher score may partly reflect headache symptoms and should not be interpreted as evidence of a specific sleep disorder.

Behavioral Problems scores were higher in both headache groups than in healthy controls. Heng et al. linked greater sleep disturbance in children with migraine to more behavioral difficulties [24]. Bursztein et al. reported more internalizing problems in children with headache [25]. Rabner et al. also found associations of sleep disturbance with anxiety, depression, and disability across headache diagnoses [13]. These findings provide context for our behavioral-domain result, but its cause cannot be identified from PSQ responses. The domain includes inattention and hyperactivity items that overlap with ADHD symptoms. Because anxiety, depression, and ADHD were not assessed systematically across the study groups, and controls with diagnosed psychiatric disorders were excluded, emotional or neurodevelopmental differences may contribute to the observed PSQ score differences. The higher behavioral and total scores therefore cannot be attributed to headache alone.

The combined headache group had a modestly higher median Breathing Problems score than controls (2 vs. 1.5), but the three-group comparison was not statistically significant, and Snoring Problems scores were similar across groups. Vendrame et al. identified sleep-disordered breathing in children with migraine who underwent polysomnography [26], while Armoni Domany et al. reported frequent snoring and obstructive sleep apnea among children with migraine referred to sleep clinics [27]. Işık et al. also found snoring to be more frequent among schoolchildren with migraine [28]. Differences in referral setting and assessment method may contribute to these contrasting findings. Because we did not systematically assess upper-airway risk factors or perform polysomnography, similar group-level snoring scores cannot exclude sleep-disordered breathing in individual children.

Other sleep disorders also fall outside this PSQ comparison. Altın et al. reported restless legs syndrome in 25.3% of children with migraine, 12.2% of those with TTH, and 8.8% of controls [29]. Restless legs symptoms and periodic limb movements were not specifically assessed in our cohort, so their contribution to the reported sleep symptoms remains unknown.

In sex-stratified analyses, PSQ total scores were higher in both female and male headache groups than in their respective controls. Among patients with migraine, age was positively correlated with PSQ total and Breathing Problems scores in females, while neither correlation was statistically significant in males. Bursztein et al. reported that sex moderated the relationship between headache and sleep [25], and Rabner et al. identified older age as a predictor of greater sleep disturbance in a pediatric headache cohort [13]. Voci et al. reported that age-related patterns varied across sleep domains [8]. Our subgroup findings are exploratory: a significant correlation in females and a nonsignificant correlation in males do not, by themselves, establish a difference between sexes. The small subgroups and lack of pubertal-stage and menstrual data further limit interpretation.

In an exploratory comparison within the headache cohort, PSQ-positive screens were recorded in 62.9% of participants assessed during a school term and 48.5% assessed during a scheduled break (odds ratio, 1.80; 95% CI, 0.78–4.15; *p* = 0.201). The estimate was imprecise, and assessment at a single visit cannot show how an individual child’s sleep changes between school terms and breaks. Differences in the patients attending during each period may also have influenced this comparison.

Our finding of more parent-reported sleep-related symptoms in children with headache does not establish which condition came first. Basile et al. discussed shared neural and circadian mechanisms, as well as possible reciprocal influences of disrupted sleep and recurrent headache [6]. In our cross-sectional study, sleep symptoms could precede headache, follow recurrent attacks, or arise alongside headache through shared factors; the available data cannot distinguish among these explanations.

Clinically, these findings support asking about sleep during the assessment of both migraine and episodic TTH. A positive PSQ screen should lead to a more specific history of sleep timing, daytime sleepiness, snoring, mood and attention symptoms, and the circumstances of any daytime naps. The questionnaire result alone should not determine a sleep-disorder diagnosis or treatment.

This study has several strengths, including headache diagnoses established by a pediatric neurologist according to ICHD-3 criteria, inclusion of both migraine and episodic TTH, an age-comparable healthy control group, use of the same validated Turkish PSQ framework across groups, and domain-level and sex-stratified analyses. Several limitations should also be acknowledged. First, the cross-sectional design does not permit causal conclusions. Second, patients were recruited from a pediatric neurology clinic whereas controls were recruited from a school, creating potential referral and selection bias; the grade-based control sampling may also have narrowed the control age distribution. An age-range-restricted sensitivity analysis was performed to assess the influence of this difference; however, restriction to the control group’s observed age range does not constitute individual age matching or eliminate residual age-related confounding. Third, sleep symptoms were parent-reported and were not corroborated by child report, sleep diaries, actigraphy, or polysomnography. Fourth, body mass index, pubertal stage, socioeconomic status, caffeine and screen exposure, adenotonsillar disease, allergic or respiratory conditions, and detailed medication use were not included in the analyses. Although visits were categorized by school-calendar period, individual school schedules and sleep timing were not assessed. Fifth, anxiety, depression, ADHD, and headache-related disability were not systematically assessed in the headache group using diagnostic interviews or standardized instruments. Although controls with a diagnosed psychiatric disorder were excluded, comparable psychiatric screening was not performed across groups. Psychiatric and neurodevelopmental comorbidity could therefore confound or amplify the observed differences in PSQ scores, particularly in the Behavioral Problems domain. The results cannot isolate an association attributable to headache itself, and the magnitude of the group difference may not generalize to the wider pediatric population. Sixth, the episodic TTH group was small, particularly after stratification by sex, and the study was not powered for migraine-subtype analyses. Seventh, multiple domain and subgroup comparisons raise the possibility of type I error despite adjusted post hoc testing, and no multivariable model was used to account for the sex imbalance between the headache and control groups. Finally, although the ADS label was selected to improve readability, the psychometric properties of this renamed domain were not independently reevaluated.

## 5. Conclusions

In this clinic-based sample, children and adolescents with migraine or episodic TTH had higher parent-reported PSQ scores than the selected school controls, particularly in the awakening/developmental and behavioral domains. Because psychiatric and neurodevelopmental comorbidities were not assessed comparably across groups, these differences cannot be attributed to headache alone. The similar total symptom burden in migraine and episodic TTH suggests that sleep screening should not be limited to migraine. The female-migraine age associations and the domain-specific respiratory findings are potentially informative but require confirmation with larger samples, objective sleep measures, and longitudinal designs. Integrating sleep assessment into pediatric headache care may help identify modifiable comorbidities and patients who require more detailed sleep evaluation.

## Figures and Tables

**Table 1:** Demographic characteristics and Pediatric Sleep Questionnaire scores in participants with primary headache disorders and healthy controls.

Characteristic	Primary Headache Disorders (*n* = 103)	Healthy Controls (*n* = 94)	*p* Value
Age, years	13.1 ± 2.44	13.1 ± 1.00	0.874
Female sex	68 (66.0)	45 (47.9)	0.010
Sleep problems (PSQ total score ≥ 6)	60 (58.3)	26 (27.7)	<0.001
PSQ total score	6 (4–8)	4 (2–6)	<0.001
Breathing Problems score	2 (1–3)	1.5 (1–3)	0.022
Awakening and Developmental Symptoms score	2 (1–2)	1 (0–2)	<0.001
Snoring score	0 (0–1)	0 (0–1)	0.659
Behavioral Problems score	1 (0–3)	0 (0–1)	<0.001

Data are presented as mean ± standard deviation, median (interquartile range), or number (%), as appropriate. Continuous variables were compared using the independent-samples *t* test or Mann–Whitney *U* test, and categorical variables using the Pearson chi-square test. PSQ, Pediatric Sleep Questionnaire. “Awakening and Developmental Symptoms” corresponds to the “Other Problems” factor in the validated Turkish extended PSQ; only the descriptive label was modified, without changing the items or scoring.

**Table 2:** Demographic characteristics and Pediatric Sleep Questionnaire scores according to headache diagnosis.

Characteristic	Migraine (n = 76)	Episodic TTH, (n = 27)	Healthy Controls, (n = 94)	*p* Value
Age, years	13.2 ± 2.6	12.7 ± 2.0	13.1 ± 1.0	0.499
Female sex	49 (64.5)	19 (70.4)	45 (47.9)	0.032
PSQ total score	6 (3–8)	7 (4–8)	4 (2–6)	<0.001
Breathing Problems score	2 (1–3)	2 (1–4)	1.5 (1–3)	0.064
ADS score	2 (1–2)	1 (1–2)	1 (0–2)	<0.001
Snoring score	0 (0–0)	0 (0–1)	0 (0–1)	0.728
Behavioral Problems score	1 (0–3)	2 (1–3)	0 (0–1)	<0.001

Data are presented as mean ± standard deviation, median (interquartile range), or number (%), as appropriate. Overall comparisons were performed using one-way analysis of variance, the Kruskal–Wallis test, or Pearson’s chi-square test. PSQ, Pediatric Sleep Questionnaire; TTH, tension-type headache. ADS, Awakening and Developmental Symptoms. “Awakening and Developmental Symptoms” corresponds to the “Other Problems” factor in the validated Turkish extended PSQ.

**Table 3:** Headache duration, attack frequency, attack duration, and headache intensity in the migraine and episodic tension-type headache groups.

Headache Characteristic	Migraine (n = 76)	Episodic TTH, (n = 27)	*p* Value
Duration of headache disorder, months	27.0 ± 27.6	12.7 ± 12.1	0.011
Attack frequency, attacks/month	11.1 ± 9.6	7.2 ± 6.3	0.021
Attack duration, hours	7.3 ± 8.6	5.9 ± 10.4	0.557
Headache intensity, score	6.9 ± 1.6	6.0 ± 1.8	0.013

Data are presented as mean ± standard deviation. Groups were compared using the independent-samples *t* test. TTH, tension-type headache.

**Table 4:** Sex-stratified comparison of Pediatric Sleep Questionnaire total and domain scores among the migraine, episodic tension-type headache, and healthy control groups.

PSQ Score	Migraine (n = 49)	Episodic TTH (n = 19)	Healthy Controls (n = 45)	*p* Value
**A. Female participants**				
Total score	6 (4–8) ^b^	7 (4.5–8) ^b^	4 (1–6)	0.003
Breathing Problems	2 (1–3.5)	2 (1–4)	2 (1–3)	0.149
Awakening and Developmental Symptoms	2 (1–2) ^a^	2 (1–2)	1 (0–2)	0.030
Snoring Problems	0 (0–0)	0 (0–1)	0 (0–0)	0.712
Behavioral Problems	1 (0–2) ^b^	2 (1–2.5) ^b^	0 (0–1)	<0.001
**B. Male participants**	**Migraine** **(n = 27)**	**Episodic** **TTH (n = 8)**	**Healthy** **Controls (n = 9)**	***p*** **Value**
Total score	6 (3–8) ^a^	6.5 (5–7.5) ^a^	3 (3–6)	0.020
Breathing Problems	2 (1–2.5)	1.5 (1–3.5)	1 (1–2)	0.734
Awakening and Developmental Symptoms	2 (1–2) ^a^	1 (1–1.5)	1 (0–1)	0.024
Snoring Problems	0 (0–0.5)	0 (0–0.5)	0 (0–1)	0.908
Behavioral Problems	2 (0–3)	2.5 (1–3) ^a^	0 (0–2)	0.040

Data are presented as median (interquartile range). Overall comparisons were performed using the Kruskal–Wallis test. ^a^ *p* < 0.05 and ^b^ *p* < 0.01 compared with healthy controls in the reported post hoc comparisons. PSQ, Pediatric Sleep Questionnaire; TTH, tension-type headache.

## Data Availability

The original contributions presented in this study are included in the article.

## Supplementary Materials

The following supporting information can be downloaded at: https://www.mdpi.com/article/10.3390/children13101357/s1, Table S1: Age-range-restricted sensitivity analysis of Pediatric Sleep Questionnaire outcomes. Table S2. PSQ outcomes after exclusion of patients with chronic migraine. Table S3. Parent-reported usual daytime napping by diagnostic group. Table S4. PSQ-positive screens by school-calendar period among patients with headache.

## Abbreviations

ADS	Awakening and Developmental Symptoms
ICHD-3	International Classification of Headache Disorders, Third Edition
IQR	Interquartile Range
NRS	Numerical Rating Scale
OSA	Obstructive Sleep Apnea
PSG	Polysomnography
PSQ	Pediatric Sleep Questionnaire
SD	Standard Deviation
TTH	Tension-Type Headache
