# Supplementary material for: Beyond the Headache: Sleep-Related Symptom Burden in Children and Adolescents with Migraine and Episodic Tension-Type Headache
Source: Children (Basel). 2026 Oct 6;13(10):1357. doi: 10.3390/children13101357 (PMC13702901; doi:10.3390/children13101357)
Supplement: Supplementary file 1 [file children-13-01357-s001.zip › children-4525519-supplementary.pdf]

Supplementary Table S1. Age-range-restricted sensitivity analysis of Pediatric Sleep Questionnaire outcomes.

Group	Original sample, n	Excluded by age restriction, n	Analyzed, n	PSQ total score, median (IQR)	PSQ ≥6, n (%)	OR vs. controls (95% CI)	p value
All patients with headache	103	21	82	6 (4–8)	47 (57.3)	3.51 (1.87–6.59)	<0.001
Migraine	76	16	60	6 (4–8)	34 (56.7)	3.42 (1.73–6.76)	<0.001
Episodic TTH	27	5	22	7 (4–8)	13 (59.1)	3.78 (1.44–9.89)	0.011
Healthy controls	94	0	94	4 (2–6)	26 (27.7)	Reference	—

Odds ratios and approximate 95% confidence intervals were calculated from the PSQ ≥6 counts shown. Fisher's exact test was used for each comparison with controls. PSQ, Pediatric Sleep Questionnaire; IQR, interquartile range; OR, odds ratio; CI, confidence interval; TTH, tension-type headache

Supplementary Table S2. PSQ outcomes after exclusion of patients with chronic migraine.

Outcome	Non-chronic migraine (n=67)	Episodic TTH (n=27)	Healthy controls (n=94)	Overall p value
PSQ total score, median (IQR)	6 (3–8)	7 (4–8)	4 (2–6)	<0.001
PSQ total score ≥6, n (%)	38 (56.7)	16 (59.3)	26 (27.7)	<0.001

IQR, interquartile range; PSQ, Pediatric Sleep Questionnaire; TTH, tension-type headache. Nine patients with chronic migraine were excluded from the original migraine group (n=76). The episodic TTH and control groups were unchanged.

Supplementary Table S3. Parent-reported usual daytime napping by diagnostic group.

Group	B5: Yes, n (%)	B5: No, n (%)	Total, n
Migraine	26 (34.2)	50 (65.8)	76
Episodic TTH	7 (25.9)	20 (74.1)	27
Healthy controls	11 (11.7)	83 (88.3)	94
All patients with headache	33 (32.0)	70 (68.0)	103

The combined headache row is the sum of the migraine and episodic TTH groups and was not entered separately into the three-group test. The overall three-group comparison used Pearson's chi-square test (p=0.002). Bonferroni-adjusted pairwise Fisher's exact tests yielded p=0.002 for migraine versus controls, p=0.360 for episodic TTH versus controls, and p=1.000 for migraine versus episodic TTH.

Supplementary Table S4. PSQ-positive screens by school-calendar period among patients with headache.

Diagnostic group	Assessed during school term, n	PSQ ≥6 during term, n (%)	Assessed during scheduled break, n	PSQ ≥6 during break, n (%)	p value*
Migraine	52	33 (63.5)	24	11 (45.8)	0.211
Episodic TTH	18	11 (61.1)	9	5 (55.6)	1.000
All patients with headache†	70	44 (62.9)	33	16 (48.5)	0.201

Fisher's exact test comparing assessment periods within each row.
